# Giant Cell Arteritis With Central Nervous System Vasculitis Presenting As Binocular Diplopia and Ptosis due to Third Cranial Nerve Palsy

**DOI:** 10.7759/cureus.99612

**Published:** 2025-12-19

**Authors:** João Casanova Pinto, Manuel G. Costa, Beatriz Fernandes, Carlos Ramalheira

**Affiliations:** 1 Internal Medicine, Hospital de Cascais Dr. José de Almeida, Alcabideche, PRT; 2 Faculty of Medicine and Health Sciences, Universitat de Barcelona, Barcelona, ESP; 3 Medical School, Universidade NOVA de Lisboa, Lisbon, PRT

**Keywords:** binocular diplopia, central nervous system vasculitis, giant cell arteritis (gca), temporal arteritis (ta), third cranial nerve (oculomotor nerve) palsy

## Abstract

We report the case of a woman in her 60s with no notable comorbidities presented with a four-week history of bilateral temporal headache, scalp tenderness, jaw claudication, and sporadic fever. She also reported binocular diplopia for the previous 24 hours. Examination revealed tender superficial temporal arteries (TA) and right-sided third cranial nerve palsy with ptosis. Laboratory tests showed elevated inflammation markers. Cranial and cervical computed tomography (CT) and CT angiography (CTA) were unremarkable. She was treated with a single dose of intravenous methylprednisolone, followed by oral prednisolone. Subsequent TA duplex ultrasound demonstrated artery wall thickness, and TA biopsy confirmed chronic inflammation with disruption of the internal elastic lamina, both consistent with giant cell arteritis. Cranial magnetic resonance imaging (MRI) revealed scattered punctate areas on T2-weighted fluid-attenuated inversion recovery (FLAIR) sequences, consistent with small-vessel vasculitis. Under corticosteroid treatment, the patient achieved full clinical remission at the four-month follow-up. This case illustrates an uncommon neurological presentation of giant cell arteritis with oculomotor nerve involvement, associated with findings suggestive of central nervous system vasculitis, and highlights the importance of early recognition and prompt corticosteroid treatment to prevent irreversible complications.

## Introduction

Giant cell arteritis (GCA) is the most common primary vasculitis affecting medium and large arteries in individuals over 50 years of age [1,2]. Typical clinical manifestations include headache, jaw claudication, and visual disturbances, most notably anterior ischemic optic neuropathy (AION). Ocular motor palsies, though less frequent, are well-documented complications arising from ischemia of the vasa nervorum supplying cranial nerves [3]. However, isolated third cranial nerve involvement with ptosis remains a rare and diagnostically challenging presentation, often mimicking benign microvascular cranial nerve palsies or compressive lesions [4].

Concurrent central nervous system (CNS) vasculitis in GCA is an even rarer phenomenon, with only sparse reports linking GCA to inflammatory changes in cerebral vasculature [5]. Recognizing atypical manifestations is key, as delayed treatment may result in severe and irreversible visual complications [2,6]. We report a case of GCA presenting with third cranial nerve palsy, with concurrent imaging findings suggestive of CNS vasculitis. This case highlights the challenges implicated in diagnosing GCA and underscores the importance of timely aggressive corticosteroid therapy.

This case was previously presented as an abstract/poster at the 22nd European Congress of Internal Medicine in Istanbul, Türkiye, 2024.

## Case presentation

A woman in her 60s, with no significant past medical history, presented to the emergency department with acute-onset binocular diplopia evolving over 24 hours. Over the preceding four weeks, she had experienced persistent bilateral temporal headache, scalp tenderness, jaw claudication, and intermittent low-grade fever (38.0 ºC). She denied any prior visual disturbances or other neurological deficits, such as facial numbness or hearing changes, as well as any drug intake, weight loss, or constitutional symptoms. There was no history of recent travel, tick bite, or immunosuppression.

**Figure 1:**
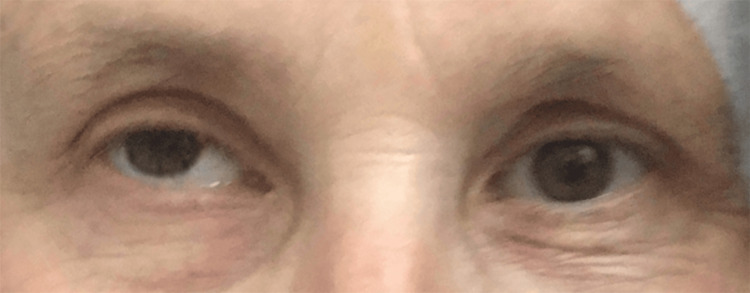
Clinical photograph demonstrating ocular misalignment (strabismus) Image shows right eye outward and upward deviation, consistent with right third cranial nerve palsy.

On admission, the patient appeared fatigued but alert and oriented. Vital signs were within normal range. Palpation of the superficial temporal arteries revealed marked tenderness and cord-like thickening bilaterally. Neurological evaluation demonstrated partial ptosis of the right eyelid with the right eye deviated outward and upward, resulting in diplopia, consistent with a right oculomotor (third cranial nerve) palsy (Figure 1). Pupillary light reflexes were intact, with no relative afferent pupillary defect. Extraocular movements were otherwise full, and fundoscopic examination did not document disc edema or pallor. The remainder of the cranial nerve examination, motor strength, sensory function, and coordination were unremarkable. The systemic examination, including peripheral pulses, bore no noteworthy findings.

Initial laboratory studies showed neutrophil-predominant leukocytosis and elevated serum inflammatory markers (Table 1).

**Table 1:** Initial laboratory tests performed ANA: antinuclear antibodies; ANCA: antineutrophil cytoplasmic antibodies; anti-CCP: anti-citrulinated peptide; C3, C4: complement components 3 and 4; CRP: C-reactive protein; ESR: erythrocyte sedimentation rate; LDL: low density lipoprotein; RF: rheumatoid factor.

Test	Patient Value	Reference range
CRP (mg/dL)	15.99	< 0.3
ESR (mm/h)	48	< 30
Leucocytes (x 10^9^/L)	12,500	4,500 – 11,000
Neutrophils (x 10^9^/L)	9,750	2,500 – 7,500
Serum creatinine (mg/dL)	0.81	0.6 – 1.1
LDL-cholesterol (mg/dL)	84	< 116
Glycosylated hemoglobin (%)	5.5	< 5.7
C3 (mg/dL)	91	80 – 180
C4 (mg/dL)	34	15 – 45
Immunological study	Negative for ANA, ANCA, RF, anti-CCP, and antiphospholipid antibodies.	

Cranial and cervical computed tomography angiography (CTA) did not reveal any aneurysms, stenosis, or occlusive arterial disease. The temporal arteries appeared symmetrically thickened, but no luminal irregularities were noted. Given the clinical suspicion for GCA with ocular involvement, high-dose corticosteroid therapy was initiated with a single dose of 250 mg intravenous methylprednisolone, followed by oral prednisolone at 40 mg daily. Within two weeks, further investigations were performed to confirm the diagnosis and exclude alternative etiologies. The metabolic panel revealed low-density lipoprotein (LDL) cholesterol of 84 mg/dL and a glycated hemoglobin of 5.5%. Blood cultures were negative.

Cerebrospinal fluid (CSF) analysis via lumbar puncture revealed normal opening pressure and absence of cytochemical abnormalities. Cytopathological evaluation, polymerase-chain reaction for Herpesviridae, microbiological cultures, autoimmunity panels, and oligoclonal bands were negative. Serological testing ruled out autoimmune mimics and infectious causes, such as syphilis, hepatitis B, hepatitis C, human immunodeficiency virus (HIV), and tuberculosis.

**Figure 2:**
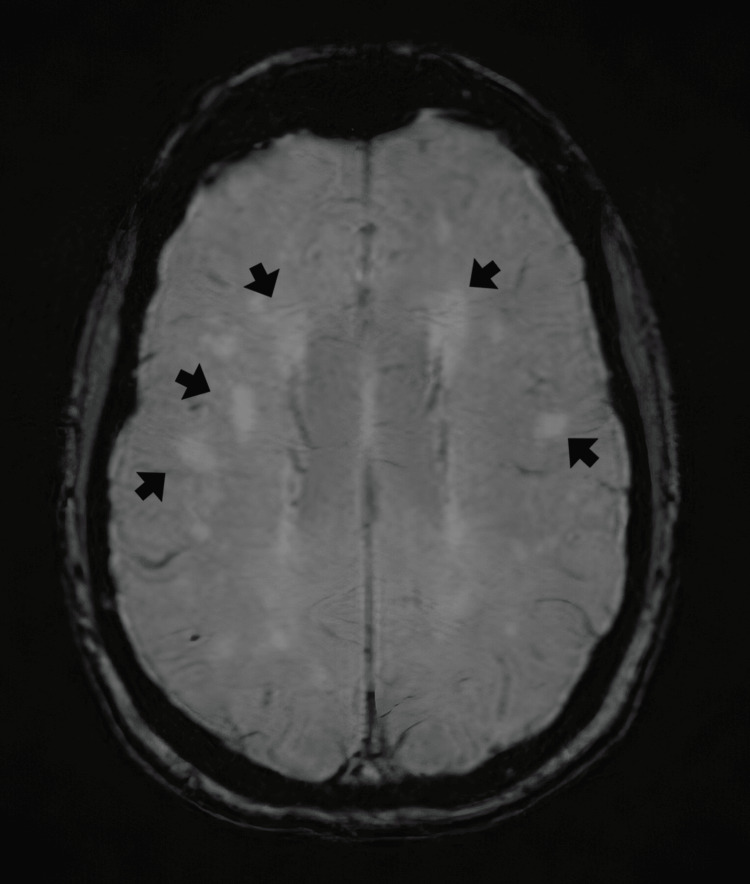
Axial T2-weighted FLAIR brain MRI image Brain MRI revealed several punctate hyperintense areas bilaterally, distributed in the frontoparietal subcortical white matter (black arrows), as well as in a periventricular distribution. These findings were interpreted as microangiopathic changes secondary to vasculitis. FLAIR: fluid-attenuated inversion recovery

A thoracic computed tomography (CT) identified a small cluster of old calcified nodules in the right upper lobe, but no evidence of active pulmonary disease. CTA ruled out supra-aortic and aortic abnormalities such as aneurysms or wall thickening. Brain magnetic resonance imaging (MRI) revealed periventricular microangiopathic chronic ischemic changes and scattered hyperintense punctate areas on T2-weighted fluid-attenuated inversion recovery (FLAIR) sequences, bilaterally distributed in the frontoparietal subcortical white matter (Figure 2). These findings were interpreted as microangiopathic changes secondary to vasculitis, with no associated contrast enhancement or mass effect.

**Figure 3:**
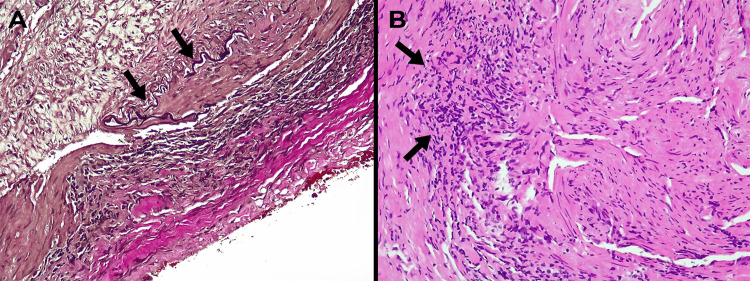
Temporal artery biopsy histology (A) Elastic stain (10×) showing marked fragmentation and disruption of the internal elastic lamina (black arrows). (B) Hematoxylin and eosin stain (20×) demonstrating dense transmural lymphohistiocytic inflammatory infiltrate involving the media (black arrows), consistent with active granulomatous arteritis.

Temporal artery duplex ultrasound demonstrated circumferential wall thickening ("halo sign") of the bilateral superficial temporal arteries, with a maximal intima-media thickness of 1.2 mm (reference value: < 0.8 mm). Subsequent temporal artery biopsy, obtained within two weeks of corticosteroid initiation, revealed a chronic inflammatory infiltrate composed predominantly of histiocytes and lymphocytes, with fragmentation of the internal elastic lamina (Figure 3).

Although granulomas and giant cells were absent, these histopathological findings, along with clinical and imaging data, satisfied 16 of the 20 points on the latest European Alliance of Associations for Rheumatology (EULAR) classification criteria for GCA [6]. The patient’s clinical course was notable for rapid improvement: within two weeks of initiating corticosteroids, her headache and jaw claudication completely resolved, and the diplopia significantly improved. Inflammatory markers progressively decreased and ultimately normalized (ESR 1 mm/h, CRP 0.2 mg/dL) by the four-month follow-up, coinciding with a full recovery of right third cranial nerve function.

A structured corticosteroid taper was planned for a total of 52 weeks, considering the one used in the giant cell arteritis Actemra (GiACTA) trial [7]. Prophylactic therapy with alendronate (70 mg/week), vitamin D (2000 IU/day), pantoprazole (40 mg/day), and cotrimoxazole (960 mg thrice/week) was initiated to mitigate glucocorticoid-induced complications. After one year, the patient experienced no relapses of the disease and is currently under prednisolone 5 mg/day. Cranial MRI at the six-month follow-up demonstrated the absence of the initially detected lesions.

## Discussion

This case illustrates two rare and clinically significant facets of GCA: third cranial nerve palsy with ptosis and concurrent CNS vasculitis. GCA is a granulomatous vasculitis of medium to large arteries, most often involving branches of the external carotid artery [1,2]. Typical symptoms include headache, mandibular claudication, and visual impairment. Among large-vessel vasculitis, GCA is the leading diagnosis in our patient, supported by her age and classic clinical features [6].

A normal CTA does not exclude GCA, which underscores the importance of temporal artery biopsy as the diagnostic gold standard, despite occasional false negatives, especially if corticosteroid treatment has already commenced [8,9]. Our patient had a positive biopsy despite being under corticosteroids for two weeks. Although visual impairment, typically from AION, remains a well-known complication, ocular cranial nerve palsies may also occur [2,3,9]. While diplopia is present in up to 19% of GCA cases, ptosis from isolated oculomotor nerve involvement is exceptionally uncommon, representing fewer than 2% of reported presentations [8]. Cranial nerve palsies in GCA are thought to result primarily from ischemia of the vasa nervorum or of small arterial branches supplying the nerve, though direct inflammatory involvement of the intracranial vasculature may also play a role [2-4]. In the presence of suspected cranial nerve involvement, brain MRI may show microangiopathic or inflammatory changes in the brain parenchyma, lending further support to the presence of a widespread vasculitis [3].

The confirmation of CNS involvement, although not routine, emphasizes the heterogeneity of GCA presentations [3,10]. In our patient, the frontoparietal T2-FLAIR hyperintense lesions were not confined to the typical periventricular or deep white matter distributions usually associated with chronic hypertensive or age-related small-vessel disease. Instead, the lesions showed a multifocal cortical-subcortical pattern, which, in conjunction with elevated inflammatory markers, cranial neuropathy, and rapid response to immunosuppression, favors an inflammatory vasculitic etiology over isolated chronic microangiopathy [11]. Remarkably, the absence of contrast enhancement or mass effect distinguishes these changes from alternative etiologies such as demyelination, neoplasm, or infectious encephalitis.

The coexistence of GCA and CNS vasculitis remains poorly understood. Both conditions involve inflammation of blood vessels, but they typically affect different vascular beds and have distinct clinical presentations. While GCA primarily involves large and medium-sized cranial arteries, CNS vasculitis affects smaller blood vessels of the brain and spinal cord. However, there are cases where these conditions overlap, suggesting potential shared mechanisms. Both GCA and CNS vasculitis are thought to arise from dysregulated immune responses. In GCA, dendritic cells in the arterial wall activate T-cells (particularly T helper 17 and T helper 1 cells), leading to the production of pro-inflammatory cytokines such as interleukin-6, interleukin-17, and interferon-gamma. These cytokines can promote systemic inflammation, which might extend to the CNS vasculature, triggering or exacerbating CNS vasculitis. The breakdown of the blood-brain barrier (BBB) due to systemic inflammation could allow immune cells and cytokines to infiltrate the CNS, contributing to vasculitis in the brain [12,13].

It has been hypothesized that similar antigens in the vascular walls of cranial and intracerebral arteries could be expressed and lead to cross-reactive immune responses. Molecular mimicry, where pathogens (e.g., viruses or bacteria) express antigens similar to those in human vascular tissue, could also play a role in initiating or perpetuating inflammation in both GCA and CNS vasculitis [14]. Endothelial dysfunction is a common feature in both GCA and CNS vasculitis. In GCA, inflammation leads to intimal hyperplasia and vessel occlusion, while in CNS vasculitis, endothelial injury can result in thrombosis, ischemia, or hemorrhage. Shared mechanisms of endothelial activation, such as upregulation of adhesion molecules and increased expression of pro-inflammatory cytokines, might contribute to the development of vasculitis in both vascular territories [15,16]. Genetic factors might predispose individuals to develop both GCA and CNS vasculitis. For example, human leukocyte antigen DRB1*04 and other immune-related genes may play a role in both GCA and CNS vasculitis, increasing its susceptibility. Familial cases of vasculitis and overlapping genetic risk factors for autoimmune diseases suggest a potential shared genetic basis [17].

Concerning the findings of concurrent CNS vasculitis, a broad range of potential etiologies was considered in the differential diagnosis, including infectious agents, systemic inflammatory/autoimmune diseases, drug-induced processes, neoplastic causes, and other miscellaneous conditions. Infections can trigger a vasculitic process either through direct invasion of the vessel wall or via immune-mediated mechanisms [5]. The patient lacked systemic signs of infection, and blood cultures and serologic testing were negative. Fungal and parasitic infections (e.g., aspergillosis, mucormycosis, neurocysticercosis, malaria) were excluded based on the patient’s immunocompetent status, lack of epidemiologic risk factors, and unsupportive imaging and laboratory results. The patient’s lack of systemic involvement, such as renal impairment, respiratory, joint, or mucocutaneous involvement make the diagnosis of other systemic inflammatory/autoimmune diseases unlikely. Similarly, negative autoantibodies, normal complement values, and absence of other specific radiological findings support GCA-associated vasculitis. In this patient, there was no history of exposure to medications or illicit substances, rendering a drug-induced etiology unlikely.

Paraneoplastic vasculitis associated with hematologic malignancies or solid tumors can occasionally present with vasculitic features [5]. However, a comprehensive evaluation provided no evidence of an underlying neoplastic process in this patient. Additional considerations include reversible cerebral vasoconstriction syndrome (RCVS) and Moyamoya disease. RCVS typically manifests as non-inflammatory vasoconstriction, which is inconsistent with the inflammatory changes observed. Moyamoya disease is characterized by chronic progressive occlusive changes and was not compatible with the acute clinical presentation and imaging findings. Collectively, the synchronized onset of systemic inflammation, cranial neuropathy, and MRI abnormalities strongly supports a unifying diagnosis of GCA with associated CNS involvement, although the absence of histopathological confirmation of cerebral vasculitis (e.g., via brain biopsy) limits definitive attribution.

When dealing with GCA, early recognition and timely administration of high-dose corticosteroids are paramount to prevent permanent visual loss, stroke, or other complications [2,18]. In this case, early initiation of high-dose intravenous methylprednisolone followed by a structured oral prednisolone taper, in accordance with recommendations for high-risk GCA, resulted in rapid clinical improvement, with complete resolution of headache and jaw claudication and marked improvement of diplopia within two weeks of treatment initiation. Inflammatory markers progressively declined and fully normalized by the four-month follow-up (ESR 1 mm/hour, CRP 0.2 mg/dL), coinciding with complete recovery of right third cranial nerve function. This favorable parallel clinical and biochemical evolution is consistent with previous reports and underscores the benefit of aggressive early immunosuppressive therapy in preventing irreversible ischemic complications [2,8]. Although adjunctive steroid-sparing agents such as tocilizumab may be considered, especially in refractory cases, they were not required in this instance [19]. Close clinical and laboratory follow-up is essential to monitor treatment response and taper regimens judiciously [18,19].

## Conclusions

This case illustrates a rare presentation of GCA manifesting as third cranial nerve palsy with ptosis and concurrent CNS vasculitis. Clinicians should maintain a high index of suspicion for GCA in older adults with an abrupt onset of diplopia, even when AION is absent. Prompt recognition, thorough diagnostic workup, and immediate initiation of high-dose corticosteroid therapy are essential to prevent severe complications, most importantly permanent vision loss. Particularly, the association between GCA and findings suggestive of CNS vasculitis is exceptionally rare, further contributing to the uniqueness of this case. Further research is needed to clarify the prevalence and prognostic implications of CNS vasculitis in GCA.
